# Human Blood Platelets Adsorption on Polymeric Materials for Liquid Biopsy

**DOI:** 10.3390/s22134788

**Published:** 2022-06-24

**Authors:** Cristina Potrich, Francesca Frascella, Valentina Bertana, Mario Barozzi, Lia Vanzetti, Federico Piccoli, Attilio Fabio Cristallo, Natalia Malara, Candido Fabrizio Pirri, Cecilia Pederzolli, Lorenzo Lunelli

**Affiliations:** 1 Fondazione Bruno Kessler, Center for Sensors and Devices, Via Sommarive 18, Povo, 38123 Trento, Italy; cpotrich@fbk.eu (C.P.); barozzi@fbk.eu (M.B.); vanzetti@fbk.eu (L.V.); cecilia.pederzolli@fbk.eu (C.P.); 2 Consiglio Nazionale delle Ricerche, Istituto di Biofisica, 38123 Trento, Italy; 3 Department of Applied Science and Technology (DISAT), Politecnico di Torino, Corso Duca degli Abruzzi 24, 10129 Turin, Italy; francesca.frascella@polito.it (F.F.); valentina.bertana@polito.it (V.B.); fabrizio.pirri@polito.it (C.F.P.); 4 Azienda Provinciale per i Servizi Sanitari, S.Chiara Hospital, 38122 Trento, Italy; federicopiccoli@gmail.com (F.P.); attcris@bls.it (A.F.C.); 5 Servizio Trasfusionale Asl 1 Imperiese, 18100 Imperia, Italy; 6 Department of Experimental and Clinical Medicine, University of Magna Graecia, 88100 Catanzaro, Italy; nataliamalara@unicz.it; 7 Istituto Italiano di Tecnologia (IIT), Via Livorno 60, 10144 Turin, Italy

**Keywords:** platelets isolation and analysis, liquid biopsy, polymeric microdevices, biomarker content

## Abstract

Platelets are emerging as a promising source of blood biomarkers for several pathologies, including cancer. New automated techniques for easier manipulation of platelets in the context of lab-on-a-chips could be of great support for liquid biopsy. Here, several polymeric materials were investigated for their behavior in terms of adhesion and activation of human platelets. Polymeric materials were selected among the most used in microfabrication (PDMS, PMMA and COC) and commercial and home-made resins for 3D printing technology with the aim to identify the most suitable for the realization of microdevices for human platelets isolation and analysis. To visualize adherent platelets and their activation state scanning, electron microscopy was used, while confocal microscopy was used for evaluating platelets’ features. In addition, atomic force microscopy was employed to further study platelets adherent to the polymeric materials. Polymers were divided in two main groups: the most prone to platelet adhesion and materials that cause few or no platelets to adhere. Therefore, different polymeric materials could be identified as suitable for the realization of microdevices aimed at capturing human platelets, while other materials could be employed for the fabrication of microdevices or parts of microdevices for the processing of platelets, without loss on surfaces during the process.

## 1. Introduction

Significant progress in early cancer detection and monitoring has been made recently, thanks to the advent of liquid biopsy [1,2,3]. Liquid biopsy refers to the analysis of non-solid biological tissues, most commonly blood, but also other body fluids. Detailed information about tumors can be indeed obtained from liquid biopsies by isolating and analyzing circulating tumor cells or nucleic acids, tumor-derived vesicles, proteins, and metabolites [4,5,6]. Platelets are considered as another relevant liquid biopsy bio-component, playing an important role in tumorigenesis [7]. They are the second most abundant cell type in peripheral blood, routinely isolated through well-established and fast methods in clinical settings [8,9]. Platelets are anucleate cell fragments, conserving the RNA processing and protein translation machinery to process RNA transcripts. Platelets emerged recently as important repositories of potential cancer biomarkers, including several types of RNAs (mRNA, miRNA, circRNA, lncRNA and mitochondrial RNA) and proteins [10]. Several preclinical studies have highlighted their potential as a liquid biopsy source to detect various types and stages of cancer [7,11]. Tumor-derived RNAs, both in peripheral blood and in the cancer microenvironment, can be taken up and stored by platelets. Moreover, platelets can alter their RNA profile in response to interaction with cancer cells or tumor-associated biomolecules (TEPs—tumor educated platelets [12]).

In this context, a parallel progress in devices and techniques capable of successfully detecting, interpreting, and monitoring cancer biomarkers in body fluids is highly desirable. Besides standard equipment and methodologies, microfluidics methods and biosensors have been exploited [13,14,15]. Microdevices and biosensors are often made principally of polymeric materials, which are in general easy to be manipulated, low-cost and suitable for many applications. In this study, the adhesion and activation of human platelets on polymeric surfaces were investigated with the final aim of identifying their properties in terms of platelet adhesion and activation. Polymers were selected among a group of materials largely used in microfabrication with the final goal of realizing microdevices for human platelets isolation followed by analysis of their biomarkers content. The selected polymeric materials include the well-known polydimethylsiloxane (PDMS), polymethyl methacrylate (PMMA) and cyclic olefin copolymer (COC), commonly used in microsystems development [16]. PDMS is a silicone-based elastomer most often used for the fabrication of microfluidic or lab on chip systems [16,17]. Although it shows very useful properties for biological and microfluidic applications, the successful translation of PDMS-based fabrication technology to mass production is limited due to inherent batch-to-batch variations and slow processing as well as its porosity and hydrophobicity. Amorphous thermoplastic polymers including PMMA and cyclic olefin copolymers are other attractive materials for microfluidic devices. The easily scaling up fabrication process of devices realized in these materials using injection molding (low fabrication cost) represents their main attraction. In addition, materials for 3D printing technology such as SpotGP (acrylate-based photoactive resin), NOA81 (Norland Optical Adhesive 81 mercapto-ester resin), PEGDA250 (poly (ethylene glycol) diacrylate) and TEGORAD2800 (silicone polyether acrylate) have been studied. The current market for printable polymers shows that only a few are considered biocompatible, but an extensive work of biological surface analysis is still in progress.

In general, platelet adhesion and activation to surfaces occur in various steps, including initial attachment, spreading, release of granule contents and platelet aggregation, related to various cellular mechanisms of activation of protein receptors as well as surface physicochemical properties (surface smoothness, surface charge, wettability, surface tension of the materials) [18,19]. Platelet adhesion on polymers depends indeed both on biological factors related to the source, such as, for example, the presence of plasma proteins or the state of the platelet donor, e.g., if stress was present when platelets were collected [20,21], and on the peculiar characteristics of materials [22,23,24]. In particular, the presence of proteins and other plasma components is critical, for example, for blood-contacting materials, whose surfaces are often modified to improve their biocompatibility [25,26].

In this work, platelets derived from human blood samples (healthy donors) were incubated in static conditions on the different materials and different imaging techniques have been used to visualize adherent platelets and their activation state. The final purpose of this study is the screening of polymeric materials to be used for the fabrication of a microdevice for platelet manipulation. Platelet containing samples can be inserted on such a microdevice and platelets could be entrapped in a suitable chamber where they can be cultured for the necessary time. The expression of biomarkers, such as nucleic acids, proteins and exosomes, could occur in this chamber, where they can be collected and analyzed. Alternatively, the isolated platelets could be recovered from the microdevice and treated with the standard methodology for biomarkers analysis.

## 2. Materials and Methods

### 2.1. Materials

#### 2.1.1. Polymers

Polydimethylsiloxane (PDMS, from Dow Corning, Sylgard 184) was used in prepolymer:curing agent ratio 10:1. Polymethyl methacrylate (PMMA) 1.5 mm thick foil was purchased from Evonik. Cyclic Olefine Copolymer (COC) Topas^®^ was purchased from Microfluidic Chipshop in 1 mm thick sheets. Both PMMA and COC can be processed by laser etching or, as in the present work, can be shaped in the desired geometry by Computer Numerically Controlled (CNC) milling. SpotGP by SpotA materials is an optically transparent stereolithography (SL) resin to be used with both commercial and customized 3D printers. In this case, a customized SL printer by Microla Optoelectronics s.r.l. was employed. It mounts a 405 nm laser and allows for setting different parameters. Furthermore, 30 mW laser power, 1500 mm/s hatching speed, 1800 mm/s contour scan speed, 0.1 mm layer thickness, and 0.04 mm hatch spacing were employed for SpotGP discs printing. NOA81 is a UV curable, transparent urethane resin produced by Norland Products Inc. The NOA81 discs were obtained by replica molding in a PDMS mold irradiated with UV light at 10 mW/cm^2^. Polyethylene glycol diacrylate (PEGDA, Mn = 250 g/mol) was used as diacrylate reactive monomers. Phenyl bis(2,4,6–trimethylbenzoyl)-phosphine oxide (BAPO) was used as a photoinitiator. All chemical solvents and reagents were obtained from Merck Company (Darmstadt, Germany) and used as received. TEGORAD 2800 (TRAD), a PDMS acrylate oligomer, was kindly given by Evonik Industries AG (Essen, Germany). The formulations were prepared by adding a 1% of BAPO photoinitiator into acrylate monomer. The 3D printing of the resin was performed using an Asiga PICO 2 DLP-3D printer (Asiga, Alexandria, Australia), equipped with a LED light source (405 nm). After the printing process, the structures were cleaned to remove the unreacted resin and UV postcured using a mercury arc lamp Dymax ECE device in the air (5 min, light intensity 10 mW cm^−2^).

#### 2.1.2. Reagents

The following reagents were purchased from Merck Life Science S.r.l. (Milan, Italy): ethanol, DPBS, glutaraldehyde, paraformaldehyde, sucrose, Mowiol 40–80. Antihuman CD62P mouse monoclonal antibody and antihuman CD41 Alexa Fluor conjugated mouse monoclonal antibody were obtained from Abcam (Abcam, Cambridge, MA, USA), while Alexa Fluor 546 donkey anti-mouse antibody and Wheat Germ Agglutinin, Alexa Fluor™ 633 Conjugate were from Invitrogen (Invitrogen, Thermofisher, Waltham, MA, USA).

### 2.2. Preparation of Polymeric Materials

Polymeric materials were prepared as thin surfaces discs-shaped of 1 cm diameter, as summarized in Table 1. After preparation, all materials were cleaned for 5 min in an ultrasonic bath in ethanol and dryed in air. Before incubation of plateles, all materials were washed again in ethanol and dryed overnight in air under a laminar hood.

### 2.3. Preparation of Platelets

Human platelets were prepared from the buffy coats of healthy donors CPD-anticoagulated blood (Immunohaematology and Transfusion Unit, S. Chiara Hospital, Trento, Italy). Ethical approval was obtained from the Ethics Committee for the clinical experimentations of the Azienda Provinciale per I Servizi Sanitari of Trento. Buffy coats were divided in 50 mL tubes and centrifuged for 14 min at 1800× *g* and 20 °C. The surnatants, enriched in platelets, were collected in new tubes, filled with 4% sodium citate pH 7.4 for 1/10 of platelets volume, as standard methodology for platelet manipulation in our laboratory. It is known that the choice of anticoagulant can affect the measured parameters, which could differ when citrate, EDTA, or other anticoagulants are used. In particular, the use of EDTA, the anticoagulant of choice for blood cell counting, is associated with phenomena like the loss of discoid shape and the time-dependent swelling of platelets, which result in an increase of the mean platelet volume (MPV). Moreover, rare but relevant, is the phenomenon known as “EDTA-dependent pseudo-thrombocytopenia” (PTCP), which however could be induced also by other anticoagulants [27]. However, if EDTA was used in place of citrate, negligible differences in platelet counts could be possibly found. Dastjerdi and colleagues have indeed demonstrated that the variable parameter MPV could also be accurately measured with both EDTA and citrate as anticoagulants, if the analysis is performed within 1 h of sampling [28]. Here, platelets were prepared just before use. The obtained platelets were counted with a Buerker chamber (about 5 × 10^5^ platelets/µL) [19,29] and tested for viability with the MTT assay [30] (100% viability), before being incubated on the polymeric materials. The presence of cell and debris contaminants in platelets preparations was negligible (less than 1%), as evidenced by SEM imaging and confocal microscopy (all membranes were stained with Wheat Germ Agglutinin Alexa Fluor™ 633 Conjugate). The presence of proteins and nucleic acids, instead, cannot be excluded. However, this aspect was not investigated in this work since the final purpose of the polymeric materials presented in this study is their use in microdevices fabrication for platelet manipulation. For this purpose, the platelet preparation used here is the most similar to the final sample, which will be loaded in the microdevice and will be in contact with the materials. The possible interference of plasma proteins and nucleic acids will likely be the same or very close for the two cases, i.e., plane surfaces and microdevices.

### 2.4. Platelets Incubation on Polymeric Materials

One millilitre of platelets prepared as described in the previous paragraph were incubated on the polymeric materials (discs of 1 cm in diameter, in 24-well microplates) in static conditions for 2 h at 37 °C and 5% CO_2_. After two washes with Dulbecco’s Phosphate Buffered Saline (DPBS), surfaces were prepared either for SEM analysis or for confocal imaging or for atomic force microscopy (AFM) imaging.

### 2.5. Characterization and Analysis

Raw polymeric materials were characterized by X-ray photoelectron spectroscopy (XPS) for the surface chemical composition, by contact angle (CA) for their wettability and by AFM for their roughness.

XPS analyses were performed using a Kratos Axis Ultra^DLD^ instrument equipped with a hemispherical analyzer and a monochromatic Al Kα (1486.6 eV) X-ray source, in spectroscopy mode. The emission angles between the axis of the analyzer and the normal to the sample surface was 0°. For each sample Si 2p, O 1s, C 1s, N 1s and S 2p core lines were recorded. XPS quantification was performed using the instrument sensitivity factors and the high-resolution scans. Charge compensation was achieved using a charge neutralizer located at the bottom of the electrostatic input lens system and all core levels were referenced to the C–C/C–H component at 285 eV. The quantification, reported as a relative elemental percentage, was made using the integrated area of the core lines after the Shirley background subtraction [31] and using atomic sensitivity factors. XPS data were analyzed using the software described in Speranza and Canteri [32].

Static contact angle was measured at room temperature with an in-house system. For each measurement, at least 3 drops of 2 µL each of ultrapure water were placed on the polymer surface and imaged with a CMOS camera. Images were analyzed with the software developed by Stalder et al. [33].

Platelets adherent on polymeric materials were characterized with scanning electron microscopy (SEM) to assess the platelet density and classify their activation state, with confocal microscopy (LSCM) to characterize platelets (distribution of CD41 and CD62P markers) and with AFM to confirm SEM results with a milder sample preparation.

*SEM analysis*: after incubation on materials, platelets were fixed with 2% glutaraldehyde for 1 h at room temperature and then were dehydrated in an ethanol-graded series (two steps of 10 min each in: ethanol 50%, 70%, 85%, 95% and absolute). Samples were dried overnight under a chemical hood, before being coated with gold (sputter coating, one cycle at 120 mA for 5 s). Imaging was performed with the JEOL–JSM-7401F scanning electron microscope, using a low energy electronic beam (700 eV, 10 µA) to reduce the electronic charging of the polymer surface. The beam aperture n.3 was used to obtain good surface details and lateral resolution. Samples were tilted for better imaging. Control images of material surfaces without platelets were acquired at the S. Chiara Hospital of Trento with a Quanta 200F FEG scanning electron microscope (Fei, Eindhoven, the Netherlands), operated in environmental mode. The total scanned surface area ranged from 2.2 × 10^5^ to 7.4 × 10^5^ µm^2^, while the minimum number of platelets counted ranged from hundreds to thousands.

*Confocal microscopy*: after incubation on materials, platelets were fixed with paraformaldehyde (PFA 2% + sucrose 2%) for 15 min at room temperature. After four washes with DPBS, platelets were double labelled as follows: materials were first incubated with antihuman CD62P mouse monoclonal antibody, diluted 1:100 in DPBS for 60 min at room temperature, washed four times with DPBS and then stained with Alexa Fluor 546 donkey anti-mouse antibody diluted 1:100 for 60 min. Finally, the samples were incubated with antihuman CD41 Alexa Fluor 488 conjugated mouse monoclonal antibody diluted 1:300 in DPBS for 60 min. In some experiments, platelets’ membranes were also stained by incubating Wheat Germ Agglutinin Alexa Fluor™ 633 Conjugate diluted 1:1000 in DPBS. After staining, samples were washed in DPBS and ultrapure water and finally mounted with one drop of Mowiol on microscope slides [34].

A Leica SP5-II confocal microscope (Leica Instruments, Wetzlar, Germany), equipped with argon (488 nm) and helium/neon lasers (543 and 633 nm), was used for imaging platelets on the polymeric materials. All samples were observed utilizing a 63× oil immersion objective: for the Alexa Fluor 546 (CD62P marker) acquisition, fluorophores were excited with the helium/neon laser, using an emission detector wavelength range from 565 to 635 nm (red channel in the following images), while, for the acquisition of Alexa Fluor 488 dye (CD41 marker, green channel in the following images), the 488-nm argon laser line was used, acquiring data in the 510–550 nm spectral region. The Alexa Fluor 633 dye was excited using the 633 nm He-Ne laser, setting the emission acquisition bandwidth from 650 to 750 nm. At least three biological replicates were measured and several images and several regions of each image were acquired. All images were analyzed with the Fiji software [35]. The CD62P marker was not quantified since its expression was clearly visible by confocal microscopy, but its mobilization toward platelet membranes attesting platelet activation was not so clear. The distinction among CD62P in the internal granules and on membranes resulted in being very difficult and almost impossible to quantify in a precise and reliable way.

*Atomic force microscopy:* after incubation on materials, platelets were fixed with paraformaldehyde (PFA 2% + sucrose 2%) for 15 min at room temperature, washed twice in DPBS and one in ultrapure water. Finally, samples were dried overnight at room temperature. AFM acquisitions were performed with a Cypher AFM equipped with an environmental scanner (Asylum Research, Santa Barbara, CA, USA) in AC mode in air at a temperature of 25 °C. Olympus OMCL-AC240TS silicon probes with a nominal force constant of 2 N/m and resonant frequency of ~70 kHz were used, acquiring ~40 fields of 30 × 30 μm^2^ for each material. AFM data were loaded and rendered with ImageJ [36], using home written plugins, and analyzed using the same software.

*Optical microscopy*: platelets prepared with the protocol for SEM, confocal and AFM analysis (see above) were measured by optical microscopy. In particular, samples prepared for SAM and AFM analysis were imaged with an optical microscope (Olympus BX51M metallurgical microscope, 50× objective), equipped with a Canon EOS 1100D camera. Samples prepared for confocal analysis were imaged with the fluorescence microscope Leica DMLA (Leica Microsystems, Wetzlar, Germany), equipped with a mercury lamp and fluorescence filter L1 (Leica Microsystems, Wetzlar, Germany). Fluorescence images were acquired with a 20× magnification objective and measured with a cooled CCD camera (DFC 420C, Leica Microsystems, Wetzlar, Germany).

## 3. Results and Discussion

Several polymeric materials commonly used in microfabrication were tested for the adhesion and activation of human platelets on their surfaces, with different imaging techniques. SEM analysis was performed to assess the platelet density and their morphology, while confocal microscopy was employed to characterize platelets in terms of specific markers (CD41 as platelets marker and CD62P as activated platelets marker). AFM measurements, instead, were conducted on PMMA, NOA81, COC and SpotGP, in order to assess the results with a milder sample preparation. PDMS, PEGDA and TG resulted in not being suitable for AFM imaging of platelet incubated samples. Finally, platelets prepared for the different imaging techniques were observed with a common technique, i.e., optical microscopy to compare results of the different preparations and acquire data on larger sample areas.

### 3.1. Materials Characterization

The physicochemical properties of the studied materials were investigated in terms of surface chemistry by XPS, in terms of wettability by contact angle and in terms of morphology by AFM. The chemical composition of polymers (Table 2) reflects in general their expected characteristics, apart from negligible contaminations. The XPS quantification is indeed in line with the literature for PMMA [37,38], PDMS [39] and NOA81 [40], while COC presents slightly more oxygen than expected [37,41]. PEGDA shows the classical carbon and oxygen content [42,43] but also a non-negligible presence of silicon. Finally, XPS data related to SpotGP and TG are difficult to compare to the literature or to the theoretical composition, since the specific chemical composition of these materials is not public or diffused by the proprietary company.

Besides the surface chemical composition, polymers were also tested for their wettability and roughness (Table 3). The highest contact angle values were found for PDMS and TG, while intermediate values were measured for PEGDA, COC and PMMA, and the lowest values were observed for SpotGP and NOA81. As observed for XPS data, CA results are in line with the literature for PDMS [44,45], COC [37,46], PMMA [37], NOA81 [40] and PEGDA [43,47], while no data are available for SpotGP and TG.

The material characterization was completed with the morphological analysis of the pure polymers. All materials were measured, a part from PDMS and PEGDA which resulted in being too soft for an accurate analysis. As for XPS and CA measurements, the surface roughness of the polymeric materials was in general good agreement with data reported in the literature, such as for COC [41,46] and PMMA [38], while, for NOA81, a slightly lower roughness is reported [40]. Again, no data for SpotGP and TG are available for comparison.

### 3.2. Characterization of Platelets with SEM

**Figure 1:**
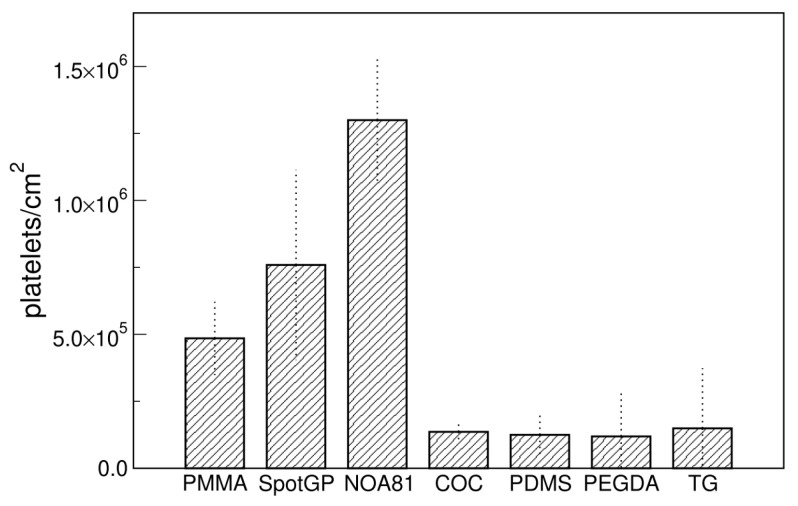
Platelets density on different materials measured with SEM. Data are means of at least four images and standard deviations are shown.

The adhesion of platelets on the seven polymeric materials analyzed in this study was firstly evaluated as density of platelets, calculated as absolute number per unit area. Comparing the different materials (Figure 1), the presence of two main groups is evident: a first group includes PMMA, SpotGP and NOA81, while the second group comprises all the four remaining materials, i.e., COC, PDMS, PEGDA and TG. The first group is characterized by a quite high platelets adhesion, which reaches (1.2 ± 0.2) × 10^6^ platelets/cm^2^ for NOA81, whereas the second group shows an average adhesion nearly 10 times lower. Polymers belonging to the two groups do not share apparently similar features, since they are prepared with different methods (PMMA by milling from sheets, SpotGP by 3D printing, NOA81 by replica molding) and are different for physical properties like stiffness, roughness as well as chemical properties, as described in the previous paragraph. PMMA is indeed a transparent thermoplastic polymer belonging to engineering plastics, while SpotGP is an acrylate-based photoactive resin, and NOA81 is a mercapto-ester resin (see Table 1).

**Figure 2:**
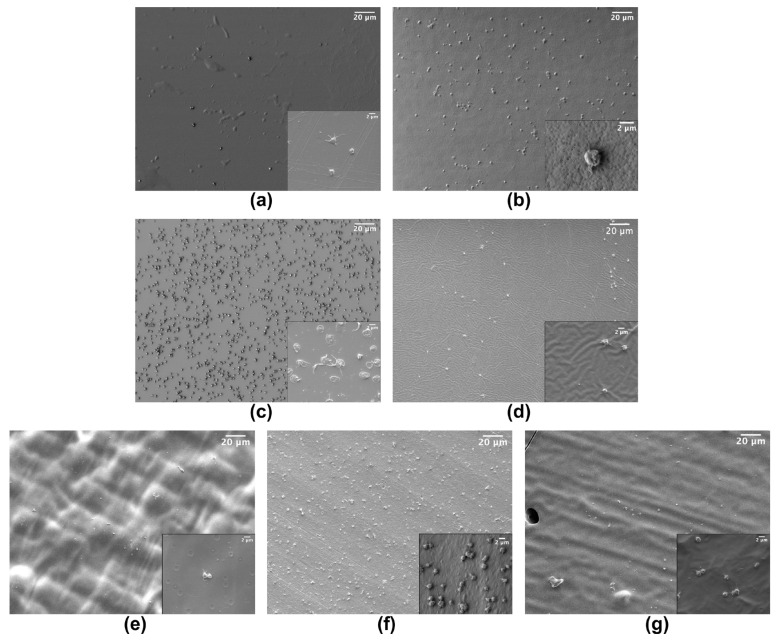
Example of SEM images of platelets adherent to: (**a**) COC; (**b**) PMMA; (**c**) NOA81; (**d**) PDMS; (**e**) PEGDA; (**f**) SpotGP and (**g**) TG. Insets are magnifications of each material (scale bar 2 µm).

Concerning platelets activation, all the tested materials showed a quite low percentage of fully spread platelets, the most common states being round and dendritic (Figure 2). In fact, platelet distribution and morphology vary on the different polymeric materials. In particular, looking at the polymers inducing the lowest adhesion, i.e., COC, PDMS, PEGDA and TG, the morphology of adherent platelets seems quite different with a similar number of platelets per unit area as well as for polymers inducing the adhesion of higher numbers of platelets. Control SEM imaging was also performed, checking for the nude materials in order to facilitate the identification of platelets.

**Figure 3:**
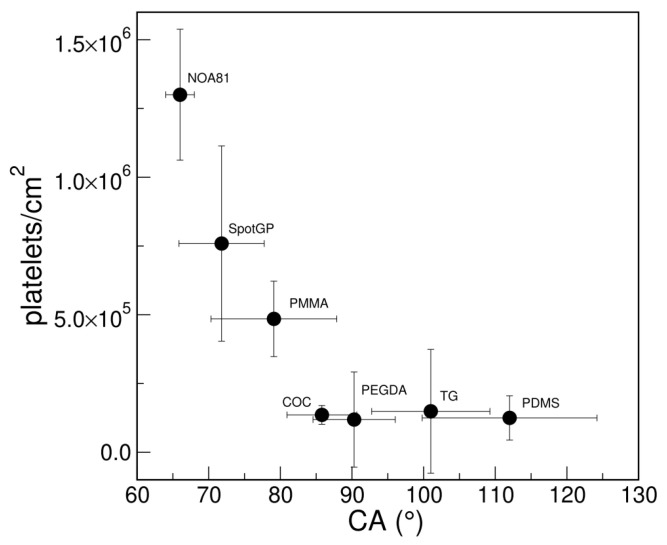
Correlation between platelet adhesions measured with SEM (density as platelets/cm^2^) and wettability (CA) of polymeric materials.

Looking at the wettability of the studied polymers, a good correlation is observed between platelet density and CA results (Figure 3). Hydrophobic surfaces, such as PDMS and TG, as well as the slightly hydrophobic polymers COC and PEGDA, show a low platelet density measured by SEM, while less hydrophobic (i.e., PMMA and SpotGP) and more hydrophilic (NOA81) polymers can capture a higher number of platelets per unit area. Therefore, the wettability of the polymeric materials resulted in being crucial both in reducing or promoting platelet adhesion.

To understand better the platelets behavior on the different materials, two other techniques were employed to image samples, i.e., confocal microscopy and atomic force microscopy.

### 3.3. Characterization of Platelets with Confocal Microscopy

**Figure 4:**
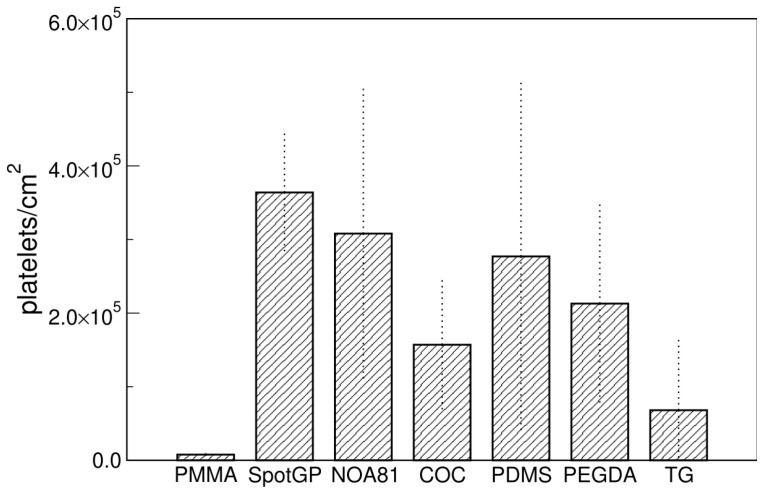
Platelets density measured by confocal microscopy. Data are means of at least three images and standard deviations are shown.

Confocal microscopy was used to evaluate the adhesion of platelets on materials and the expression of two platelet markers, often used to define platelets, i.e., CD41, as marker peculiar of platelets [48,49] and CD62P (P-selectin) as a marker of activated platelets, playing a critical role in platelet aggregation [49,50,51]. Concerning platelets adhesion, the density of platelets was calculated by counting all platelets thanks to the staining of CD41 antigens and membranes (Figure 4). A lower amount of platelets was observed on all samples with respect to SEM imaging, with no evident stratification of materials, except for the very low adhesion found on PMMA. SpotGP and NOA81 were confirmed as prone to platelets adhering, but similarly to PDMS and, less, to PEGDA. The last three materials mentioned showed a quite high variability among biological replicates, as attested by the quite large errors shown in Figure 4. Moreover, COC and TG were confirmed as low-capturing materials, while PMMA was, surprisingly and opposite to SEM results, the polymer with the lowest platelets density.

**Figure 5:**
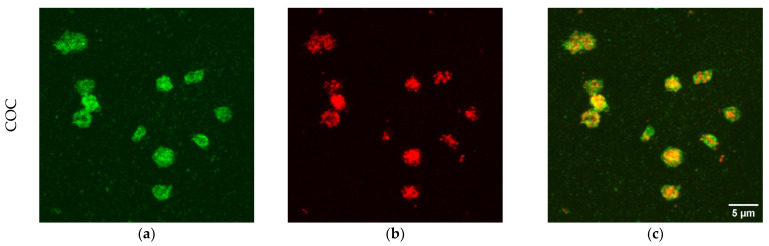

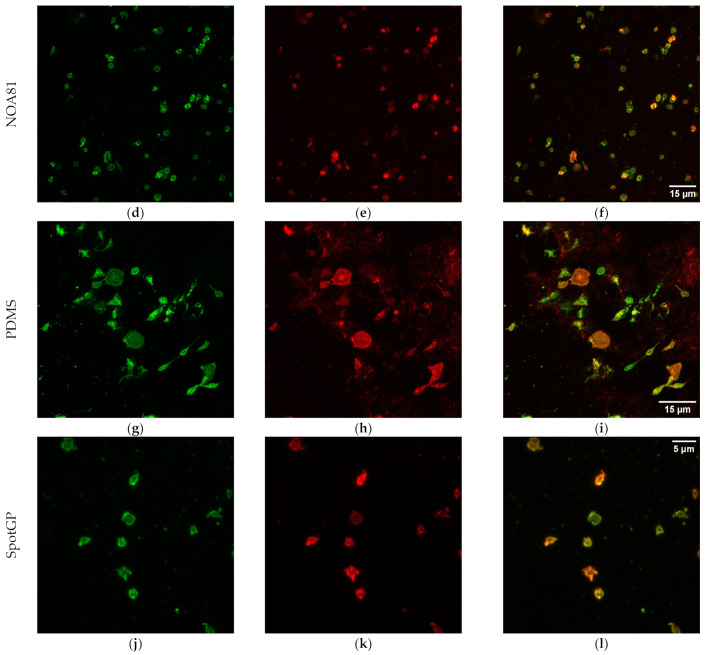
Confocal images of platelets adherent to polymeric materials. In the first column, the signal of CD41 is reported (panels (**a**,**d**,**g**,**j**)), while, in the second, the signal of CD62P is shown (panels (**b**,**e**,**h**,**k**)). The last column refers to merged signal (co-localization, (**c**,**f**,**i**,**l**)). Scale bars: 5 µm for COC and SpotGP, 15 µm for NOA81 and PDMS.

Besides number of platelets per unit area, the presence of platelets markers was evaluated. The CD41 antigen was selected as a platelet marker, while CD62P was selected as a marker of activated platelets. CD41 is a transmembrane glycoprotein, alternatively named integrin αIIb, known as a marker of the megakaryocytic lineage [48,52]. CD62P, or P-selectin, is an established indicator of platelet activation that is usually stored in platelet α-granules, but upon appropriate activation is rapidly mobilized to the platelet membrane [50,51], where the expression of CD62P is dramatically increased. This increment is accompanied by a simultaneous increase in the expression level of plasma soluble CD62, which plays an important role in the initiation, formation, and expansion of thrombi. Beside platelet markers, membranes were also stained to demonstrate the possible presence of blood cells beyond platelets. Typical examples of platelets imaged on the different polymeric materials are shown in Figure 5, but all materials were imaged in the same conditions (see Figure S1 of the Supplementary Materials). In the first column of the figure the channel of CD41 is reported, in the second column, the channel of CD62P, while, in the third column the merge of the two channels is reported, to facilitate the marker co-localization. The CD41 antigen is homogeneously distributed on the membrane of platelets adherent on all four materials shown in the first column of Figure 5, as expected for a marker that identifies platelets. On the contrary, the distribution of CD62P varies on the different polymers (see the second column in Figure 5). Platelets adherent to COC present CD62P as granules inside the cell, not mobilized to the membrane, as visible from the co-localization image (third column in Figure 5). This material induces the adhesion of few platelets, as observed in absolute number per unit area both from SEM imaging (Figure 1) and from confocal microscopy (Figure 4). Moreover, the shape of the (few) platelets adherent to COC is round or discoid, typical of normal circulating platelets or to the first state in the adhesion to the surface [18,53]. On the contrary, PDMS and SpotGP show many activated platelets from dendritic and spread dendritic shape (Figure 5l) to fully spread (Figure 5i). On these materials, platelets mobilized the CD62P marker on their membranes, initiating the activation process, as attested by the co-localization of CD41 and CD62P visible in panels (i) and (l) of Figure 5. An intermediate behavior is instead observed for platelets adherent to NOA81, where round, dendritic and spread dendritic shaped platelets are visible. Analogously to shapes, CD62P distribution varies from inside the cell (localized into the granules) to the cell membrane (see panel (f) in Figure 5). Again, the studied polymeric materials are found to have different properties both in terms of platelet adhesion and activation, allowing for suggesting their use well-fitting different applications.

### 3.4. Characterization of Platelets with AFM

**Figure 6:**
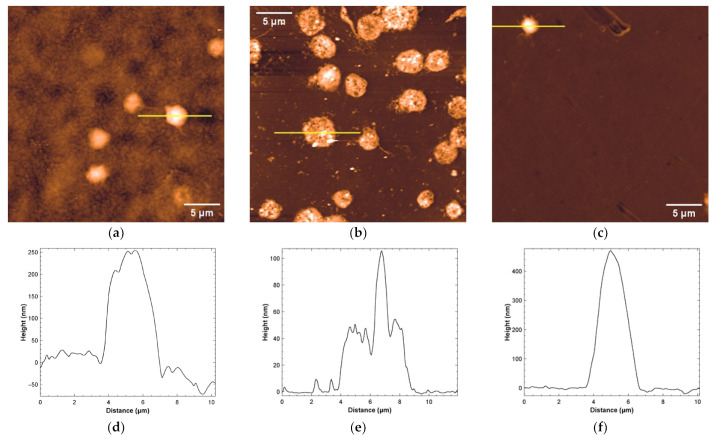
Representative AFM analysis of platelets adherent to: (**a**) PMMA (z false-color scale from −50 to 250 nm), (**b**) NOA81 (z false-color scale from −10 to 80 nm), and (**c**) COC (z false-color scale: −50 to 450 nm). The height profiles traced along the yellow lines are shown respectively in (**d**–**f**).

To compare and consolidate results obtained with different imaging techniques, AFM measurements were performed. Three polymeric materials were found suitable for AFM imaging, i.e., PMMA, NOA81 and COC. These materials are hard and not too rough and therefore suitable for this kind of analysis. A fourth material (SpotGP) also shared similar characteristics, but, after platelet incubation, a quite continuous layer was found to cover the original sample, with negligible possibility to count individual platelets (see Figure S2). The platelet density and morphology on the material surface were then analyzed (Figure 6), observing a good density on NOA81 (panel b), a low density on COC (panel c) and intermediate values for PMMA (panel a). These results nicely correlate with SEM measurements, but only partially with LSCM analysis. The platelet morphology is also clearly visible in AFM images, allowing for comparing the activation state with SEM and LSCM results (Figure 2 and Figure 5, respectively). Again, data obtained both with AFM and SEM are in good agreement. Platelets on PMMA are indeed mainly round-shaped, with a thickness ranging around hundreds of nm (Figure 6, profile in panel d), while on NOA81 they are in different activation states, also confirming data obtained with confocal microscopy (see panels (e) and (f) of Figure 6). On this material, the maximum platelet thickness reaches 100 nm (panel e). Few platelets are adherent to COC with a mainly round shape, which reach a maximum thickness of about 0.5 µm (see profile in panel (f) of Figure 6).

To explain the differences in platelet number per unit area observed with the three techniques used in this study to analyze same samples, a comparison among results was conducted (see the next paragraph).

### 3.5. Comparison among Different Imaging Techniques

**Figure 7:**
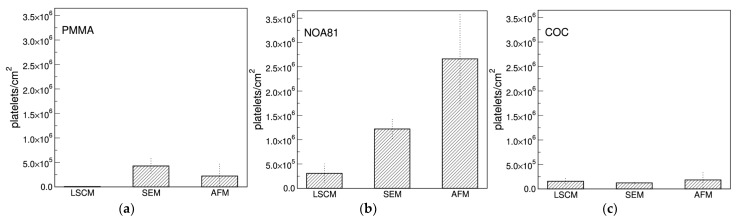
Comparison among platelets density per unit area measured with the different imaging techniques. In (**a**) platelets adherent to PMMA are shown, in (**b**) to NOA81 and in (**c**) to the COC polymer.

As shown in the previous paragraphs, different results were obtained by imaging the same samples with different techniques. The comparison of the adhesion extent using different analytical methods, indeed, highlights a quite large difference for some materials such as PMMA and, even more, NOA81 (Figure 7a,b), while others seem not very sensitive to the method of analysis (COC, Figure 7c). This behaviour may depend on the severity of the sample preparation that is needed for the different analytical techniques, the AFM being the mildest, and the LSCM requiring several incubation and washing steps for the fluorescent staining (see Section 2.5). This may indicate that the platelet adhesion on NOA81 is high, but not particularly strong, and that platelets can be then easily detached by fluxing aqueous buffers. This result could also indicate that the graph in Figure 4 and the images of Figure 5 actually refers to a platelet subpopulation that is composed by the platelets that are more strongly attached to the materials. Moreover, a similar behaviour could be found on materials which cannot be successfully examined with AFM (see Figure S3). SpotGP shows indeed the highest platelet density with a marked decrease when observed with LSCM. This phenomenon is not clearly visible for the other polymers shown in Figure S3.

To validate this comparison, a further analysis was conducted by optical microscopy on PMMA, NOA81 and COC samples prepared for LSCM, SEM and AFM. In this way, large sample areas, similar for all the three preparations, were acquired and analyzed. Samples prepared with the protocol used for SEM and AFM imaging were measured with optical microscopy, and wide field fluorescence microscopy was used for confocal preparations (these samples were impossible to be measured by optical microscopy because of the mounting medium used in the preparation). Results, reported in Figure S4 of the Supplementary Materials, confirmed the behavior observed with the different imaging methods for all the three polymers analyzed very well. Therefore, the differences in platelets density observed by the different techniques could be ascribed to the different protocols of sample preparation. In summary, the most hydrophilic materials show both the highest platelet density, alongside a substantial reduction when treated with the protocol for confocal imaging. In contrast, platelet density on more hydrophobic materials is not very sensitive to the different treatments, and may also be higher when materials are treated with the confocal imaging protocol.

This different behaviour in terms of platelets adhesion can be indeed exploited for different purposes in the design of microdevices dedicated to platelets manipulation. Polymers showing high densities of adherent platelets could be employed for the microfabrication of tools for platelet entrapping and concentration, while those showing low propensity for platelets adhesion could be devoted to the realization of connections, where a low adhesion is instead desirable, and/or to the realization of chambers working with platelets in suspension.

## 4. Conclusions

Detailed information about tumors can be obtained from liquid biopsies by isolating and analyzing circulating tumor cells or nucleic acids, tumor-derived vesicles, proteins and metabolites. Platelets are considered another relevant liquid biopsy bio-component, are abundant in peripheral blood, and can be routinely obtained in clinical settings. Platelets are emerging as important repositories of potential cancer biomarkers, including several types of RNAs and proteins, and several preclinical studies have highlighted their potential as a liquid biopsy source to detect various types and stages of cancer. In this work, seven different polymeric materials used in the microdevice fabrication were selected to study platelet adhesion and activation, finding that the adhesion of platelets can be effectively modulated by an opportune choice of materials.

The tested materials present different advantages. PDMS is commonly employed for research purposes in the field of biological analysis and microfluidic platform. Together with NOA81, they give the possibility to precisely replicate the geometry of a given mold. Good accuracy can be also achieved with 3D printable materials, as PEGDA, SpotGP and Tegorad. Moreover, they offer the possibility to build up analysis platforms in a single process. However, all these materials are often expensive if compared to thermoplastics like COC and PMMA. Even if the latter can introduce more geometry restrictions, since they are rigid, they give the possibility to produce devices not only by Computer Numerical Control (CNC) milling, but also with more scalable processes, such as embossing or injection molding. Therefore, an injection molded device in PMMA with NOA81 coating would be the most convenient combination in terms of cost-efficiency.

These results are promising for a future exploitation aimed at fabricating a microdevice for the efficient isolation of platelets in dedicated microchambers, while attaining a low attachment on surfaces of microfluidic connections [54]. A final perspective of this microdevice is the analysis of platelet biomarkers content, particularly miRNAs and exosomes.

## Figures and Tables

**Table 1:** Polymer materials tested in this study. Name, description and main features are reported.

Polymer Name	Polymer Description	Typical Use in Microfabrication	Deposition/Fabrication
COC	cyclic olefin copolymer	microsystems, low fabrication cost	CNC milling from sheets
PMMA	polymethyl methacrylate	microsystems, low fabrication cost	CNC milling from sheets
NOA81	Norland Optical Adhesive 81 (mercapto-ester resin)	bonding technology	replica molding—UV curing
PDMS	polydimethylsiloxane	prototyping, small batch production	replica molding
PEGDA	poly (ethylene glycol) diacrylate PEGDA250	3D printing technology	LED 3D printing—UV post curing
SpotGP	acrylate-based photoactive resin	3D printing technology	laser stereolithography
TG	silicone polyether acrylate TEGORAD2800	3D printing technology	LED 3D printing—UV post curing

**Table 2:** XPS analysis at 0° take-off angle of all polymers studied. The standard error does not exceed the 1–2% of the reported value.

Polymer	O 1s (%)	N 1s (%)	C 1s (%)	S 2p (%)	Si 2p (%)
COC	22.2	-	77.	0.2	0.5
PMMA	23.8	-	73.7	-	0.5
NOA81	29.7	5.6	58.4	5.4	1.0
PDMS	33.4	-	53.4	-	13.2
PEGDA	27.6		60.4	-	12.0
SpotGP	23.7	0.9	74.7	-	0.6
TG	27.1	-	60.0	-	12.9

**Table 3:** Wettability (CA) and roughness (RMS) of the polymeric materials. Standard deviations are shown.

Polymer	CA (°)	RMS (nm)
COC	85.8 ± 4.87	5.0 ± 1.0
PMMA	79.1 ± 8.8	14.6 ± 0.7
NOA81	66.0 ± 2.0	3.3 ± 1.2
PDMS	112.0 ± 12.2	-
PEGDA	90.3 ± 5.7	-
SpotGP	71.8 ± 5.9	76.0 ± 3.6
TG	101.0 ± 8.2	61.4 ± 15.1

## Data Availability

The data presented in this study are available from the corresponding author upon request.

## Supplementary Materials

The following supporting information can be downloaded at: https://www.mdpi.com/article/10.3390/s22134788/s1, Figure S1: Confocal images of platelets adherent to polymeric materials, Figure S2: Typical AFM images of (a): untreated SpotGP, (b) SpotGP after platelets incubation, Figure S3: Comparison of platelet densities observed with Scanning Electron Microscopy and Laser Scanning Confocal Microscopy, Figure S4: Comparison of platelet densities obtained with different sample preparations.

## References

[1] Castro-Giner F., Gkountela S., Donato C., Alborelli I., Qualgliata L., Ng C.K.Y., Piscuoglio S., Aceto N. (2018). Cancer Diagnosis Using a Liquid Biopsy: Challenges and Expectations. Diagnostics.

[2] Qiu J., Xu J., Zhang K., Gu W., Nie L., Wang G., Luo Y. (2020). Refining Cancer Management Using Integrated Liquid Biopsy. Theranostics.

[3] Martins I., Ribeiro I., Jorge J., Gonçalves A., Sarmento-Ribeiro A., Melo J., Carreira I. (2021). Liquid Biopsies: Applications for Cancer Diagnosis and Monitoring. Genes.

[4] Bunda S., Zuccato J., Voisin M., Wang J., Nassiri F., Patil V., Mansouri S., Zadeh G. (2021). Liquid Biomarkers for Improved Diagnosis and Classification of CNS Tumors. Int. J. Mol. Sci..

[5] Lianidou E., Pantel K. (2019). Liquid biopsies. Genes Chromosomes Cancer.

[6] Malara N., Trunzo V., Foresta U., Amodio N., de Vitis S., Roveda L., Fava M., Coluccio M., Macrì R., di Vito A. (2016). Ex-vivo characterization of circulating colon cancer cells distinguished in stem and differentiated subset provides useful biomarker for personalized metastatic risk assessment. J. Transl. Med..

[7] Antunes-Ferreira M., Koppers-Lalic D., Wuerdinger T. (2021). Circulating platelets as liquid biopsy sources for cancer detection. Mol. Oncol..

[8] Ghoshal K., Bhattacharyya M. (2014). Overview of Platelet Physiology: Its Hemostatic and Nonhemostatic Role in Disease Pathogenesis. Sci. World J..

[9] Montague S., Lim J., Lee W., Gardiner E. (2020). Imaging Platelet Processes and Function—Current and Emerging Approaches for Imaging in vitro and in vivo. Front. Immunol..

[10] Best M., Wesseling P., Wurdinger T. (2018). Tumor-Educated Platelets as a Noninvasive Biomarker Source for Cancer Detection and Progression Monitoring. Cancer Res..

[11] Sabrkhany S., Kuijpers M., Egbrink M.O., Griffioen A. (2021). Platelets as messengers of early-stage cancer. Cancer Metastasis Rev..

[12] Best M.G., Sol N., Kooi I.E., Tannous J., Westerman B.A., Rustenburg F., Schellen P., Verschueren H., Post E., Koster J. (2015). RNA-Seq of Tumor-Educated Platelets Enables Blood-Based Pan-Cancer, Multiclass, and Molecular Pathway Cancer Diagnostics. Cancer Cell.

[13] Iliescu F., Poenar D., Yu F., Ni M., Chan K., Cima I., Taylor H., Cima I., Iliescu C. (2019). Recent advances in microfluidic methods in cancer liquid biopsy. Biomicrofluidics.

[14] Kumar S., Han J., Michael I., Ki D., Sunkara V., Park J., Gautam S., Ha H., Zhang L., Cho Y. (2019). Human Platelet Membrane Functionalized Microchips with Plasmonic Codes for Cancer Detection. Adv. Funct. Mater..

[15] Razmi N., Baradaran B., Hejazi M., Hasanzadeh M., Mosafer J., Mokhtarzadeh A., de la Guardia M. (2018). Recent advances on aptamer-based biosensors to detection of platelet-derived growth factor. Biosens. Bioelectron..

[16] Nayak S., Blumenfeld N., Laksanasopin T., Sia S. (2017). Point-of-Care Diagnostics: Recent Developments in a Connected Age. Anal. Chem..

[17] Potrich C., Lunelli L., Cocuzza M., Marasso S.L., Pirri C., Pederzolli C. (2018). Simple PDMS microdevice for biomedical applications. Talanta.

[18] Goodman S. (1999). Sheep, pig, and human platelet-material interactions with model cardiovascular biomaterials. J. Biomed. Mater. Res..

[19] Forti S., Lunelli L., della Volpe C., Siboni S., Pasquardini L., Lui A., Canteri R., Vanzetti L., Potrich C., Vinante M. (2011). Hemocompatibility of pyrolytic carbon in comparison with other biomaterials. Diam. Relat. Mater..

[20] Lamponi S., Aloisi A., Barbucci R. (1999). The role of Fbg in platelet adhesion to polymeric materials in conditions of psychological stress. Biomaterials.

[21] Weber N., Wendel H., Kohn J. (2005). Formation of viscoelastic protein layers on polymeric surfaces relevant to platelet adhesion. J. Biomed. Mater. Res. Part A.

[22] Sharma C. (2001). Bolood-compatible materials: A perspective. J. Biomater. Appl..

[23] Xu W., Xiao M., Yuan L., Zhang J., Hou Z. (2018). Preparation, Physicochemical Properties and Hemocompatibility of Biodegradable Chitooligosaccharide-Based Polyurethane. Polymers.

[24] Skarja G., Brash J. (1997). Physicochemical properties and platelet interactions ofsegmented polyurethanes containing sulfonate groups inthe hard segment. J. Biomed. Mater. Res..

[25] Li P., Cai W., Li X., Wang K., Zhou L., You T., Wang R., Chen H., Zhao Y., Wang J. (2020). Preparation of phospholipid-based polycarbonate urethanes for potential applications of blood-contacting implants. Regen. Biomater..

[26] Kuźmińska A., Wojciechowska A., Butruk-Raszeja B. (2021). Vascular Polyurethane Prostheses Modified with a Bioactive Coating—Physicochemical, Mechanical and Biological Properties. Int. J. Mol. Sci..

[27] Mannuss S. (2020). Influence of different methods and anticoagulants on platelet parameter measurement. J. Lab. Med..

[28] Dastjerdi M., Emami T., Najafian A., Amini M. (2006). Mean platelet volume measurement, EDTA or citrate?. Hematology.

[29] Bürker K. (1911). über weitere Verbesserungen der Methode zur ZÄhlung roter Blutkörperchen nebst einigen ZÄhlresultaten. Pflüger’s Arch..

[30] Benedetti M., Torresani E., Leoni M., Fontanari V., Bandini M., Pederzolli C., Potrich C. (2017). The effect of post-sintering treatments on the fatigue and biological behavior of Ti-6Al-4V ELI parts made by selective laser melting. J. Mech. Behav. Biomed. Mater..

[31] Shirley D. (1972). High-Resolution X-Ray Photoemission Spectrum of the Valence Bands of Gold. Phys. Rev. B.

[32] Speranza G., Canteri R. (2019). RxpsG a new open project for Photoelectron and Electron Spectroscopy data processing. SoftwareX.

[33] Stalder A., Kulik G., Sage D., Barbieri L., Hoffmann P. (2006). A Snake-Based Approach to Accurate Determination of Both Contact Points and Contact Angles. Colloids Surf. A Physicochem. Eng. Asp..

[34] Romano G., Mancini R., Fedele P., Curigliano G., Flamini G., Giovagnoli M., Malara N., Boninsegna A., Vecchione A., Santella R. (1997). Immunohistochemical analysis of 4-aminobiphenyl-DNA adducts in oral mucosal cells of smok-ers and nonsmokers. Anticancer Res..

[35] Schindelin J., Arganda-Carreras I., Frise E., Kaynig V., Longair M., Pietzsch T., Preibisch S., Rueden C., Saalfeld S., Schmid B. (2012). Fiji: An open-source platform for biological-image analysis. Nat. Methods.

[36] Schneider C., Rasband W., Eliceiri K. (2012). NIH image to imagej: 25 years of image analysis. Nat. Methods.

[37] Tsao C., Hromada L., Liu J., Kumar P., DeVoe D. (2007). Low temperature bonding of PMMA and COC microfluidic substrates using UV/ozone surface treatment. Lab Chip.

[38] Nathawat R., Kumar A., Acharya N., Vijay Y. (2009). XPS and AFM surface study of PMMA irradiated by electron beam. Surf. Coat. Technol..

[39] Santini G., Potrich C., Lunelli L., Vanzetti L., Marasso S., Cocuzza M., Pirri C., Pederzolli C. (2017). miRNA purification with an optimized PDMS microdevice: Toward the direct purification of low abundant circulating biomarkers. Biophys. Chem..

[40] Wägli P., Homsy A., de Rooij N. (2011). Norland optical adhesive (NOA81) microchannels with adjustable wetting behavior and high chemical resistance against a range of mid-infrared-transparent organic solvents. Sens. Actuators B.

[41] el Fissi L., Vandormael D., Houssiau L., Francis L. (2016). Surface functionalization of cyclic olefin copolymer (COC) with evaporated TiO_2_ thin film. Appl. Surf. Sci..

[42] Liu L., Fei T., Guan X., Zhao H., Zhang T. (2021). Highly sensitive and chemically stable NH3 sensors based on an organic acid-sensitized cross-linked hydrogel for exhaled breath analysis. Biosens. Bioelectron..

[43] Ju J., Wang T., Wang Q. (2015). Superhydrophilic and underwater superoleophobic PVDF membranes via plasma-induced surface PEGDA for effective separation of oil-in-water emulsions. Colloids Surf. A Physicochem. Eng. Asp..

[44] Lin C., Yeh Y., Lin W., Yang M. (2014). Novel silicone hydrogel based on PDMS and PEGMA for contact lens application. Colloids Surf. B Biointerfaces.

[45] Seo J., Lee L. (2006). Effects on wettability by surfactant accumulation/depletion in bulk polydimethylsiloxane (PDMS). Sens. Actuators B Chem..

[46] Roy S., Yue C., Lam Y., Wang Z., Hu H. (2010). Surface analysis, hydrophilic enhancement, ageing behavior and flow in plasma modified cyclic olefin copolymer (COC)-based microfluidic devices. Sens. Actuators B Chem..

[47] Tan G., Chen R., Ning C., Zhang L., Ruan X., Liao J. (2012). Effects of Argon Plasma Treatment on SurfaceCharacteristic of PhotopolymerizationPEGDA–HEMA Hydrogels. J. Appl. Polym. Sci..

[48] Paul A., Straub A., Weber N., Ziemer G., Wendel H. (2009). CD41 Western blotting: A new method to detect platelet adhesion to artificial surfaces used in extracorporeal circulation procedures. J. Mater. Sci. Mater. Med..

[49] Muthusubramaniam L., Lowe R., Fissell W., Li L., Marchant R., Desai T., Roy S. (2011). Hemocompatibility of Silicon-Based Substrates for Biomedical Implant Applications. Ann. Biomed. Eng..

[50] Hegazy S., Elsabaawy M., Eltabakh M., Hammad R., Bedair H. (2021). CD62P (P-selectin) expression as a platelet activation marker in patients with liver cirrhosis with and without cholestasis. Clin. Exp. Hepatol..

[51] Shen L., Yang T., Xia K., Yan Z., Tan J., Li L., Qin Y., Shi W. (2020). P-selectin (CD62P) and soluble TREM-like transcript-1 (sTLT-1) are associated with coronary artery disease: A case control study. BMC Cardiovasc. Disord..

[52] Robin C., Ottersbach K., Boisset J., Oziemlak A., Dzierzak E. (2011). CD41 is developmentally regulated and differentially expressed on mouse hematopoietic stem cells. Blood.

[53] George J. (2000). Platelets. Lancet.

[54] Perozziello G., Simone G., Candeloro P., Gentile F., Malara N., Larocca R., Coluccio M., Pullano S., Tirinato L., Geschke O. (2010). A Fluidic Motherboard for Multiplexed Simultaneous and Modular Detection in Microfluidic Systems for Biological Application. Micro Nanosyst..

