# Supplementary material for: Human Blood Platelets Adsorption on Polymeric Materials for Liquid Biopsy
Source: Sensors (Basel). 2022 Jun 24;22(13):4788. doi: 10.3390/s22134788 (PMC9269204; doi:10.3390/s22134788)
Supplement: Supplementary file 1 [file sensors-22-04788-s001.zip › sensors-1691844-supplementary.pdf]

sensors

Supplementary Information

Human blood platelets adsorption on polymeric materials for liquid biopsy

Cristina Potrich [1,2], Francesca Frascella[3], Valentina Bertana[3], Mario Barozzi[1], Lia Vanzetti[1], Federico Piccoli[4], Attilio Fabio Cristallo[4], Natalia Malara[5], Candido Fabrizio Pirri[3,6], Cecilia Pederzolli [1]and Lorenzo Lunelli [1,2,*]

1 Fondazione Bruno Kessler, Center for Sensors and Devices, Via Sommarive 18, Povo, I-38123 Trento, Italy; cpotrich@fbk.eu (C.Po.); lunelli@fbk.eu (L.L.); barozzi@fbk.eu (M.B.); vanzetti@fbk.eu (L.V.); cecilia.pederzolli@fbk.eu (C.Pe.)
2 Consiglio Nazionale delle Ricerche-Istituto di Biofisica, Trento, 38123, Italy
3 Department of Applied Science and Technology (DISAT), Politecnico di Torino, Corso Duca degli Abruzzi 24, 10129 Torino, Italy; francesca.frascella@polito.it (F.F.); valentina.bertana@polito.it (V.B.); fabrizio.pirri@polito.it (C.F.P.)
4 Azienda Provinciale per i Servizi Sanitari, S.Chiara Hospital, 38122 Trento, Italy; federico.piccoli@apss.tn.it (F.P.); attiliofabio.cristallo@apss.tn.it (A.F.C.)
5 University of Magna Graecia, 88100 Catanzaro, Italy; nataliamalara@unicz.it (N.M.)
6 Istituto Italiano di Tecnologia (IIT)Via Livorno 60, Turin 10144, Italy
* Correspondence: lunelli@fbk.eu; +39 0461 314 604

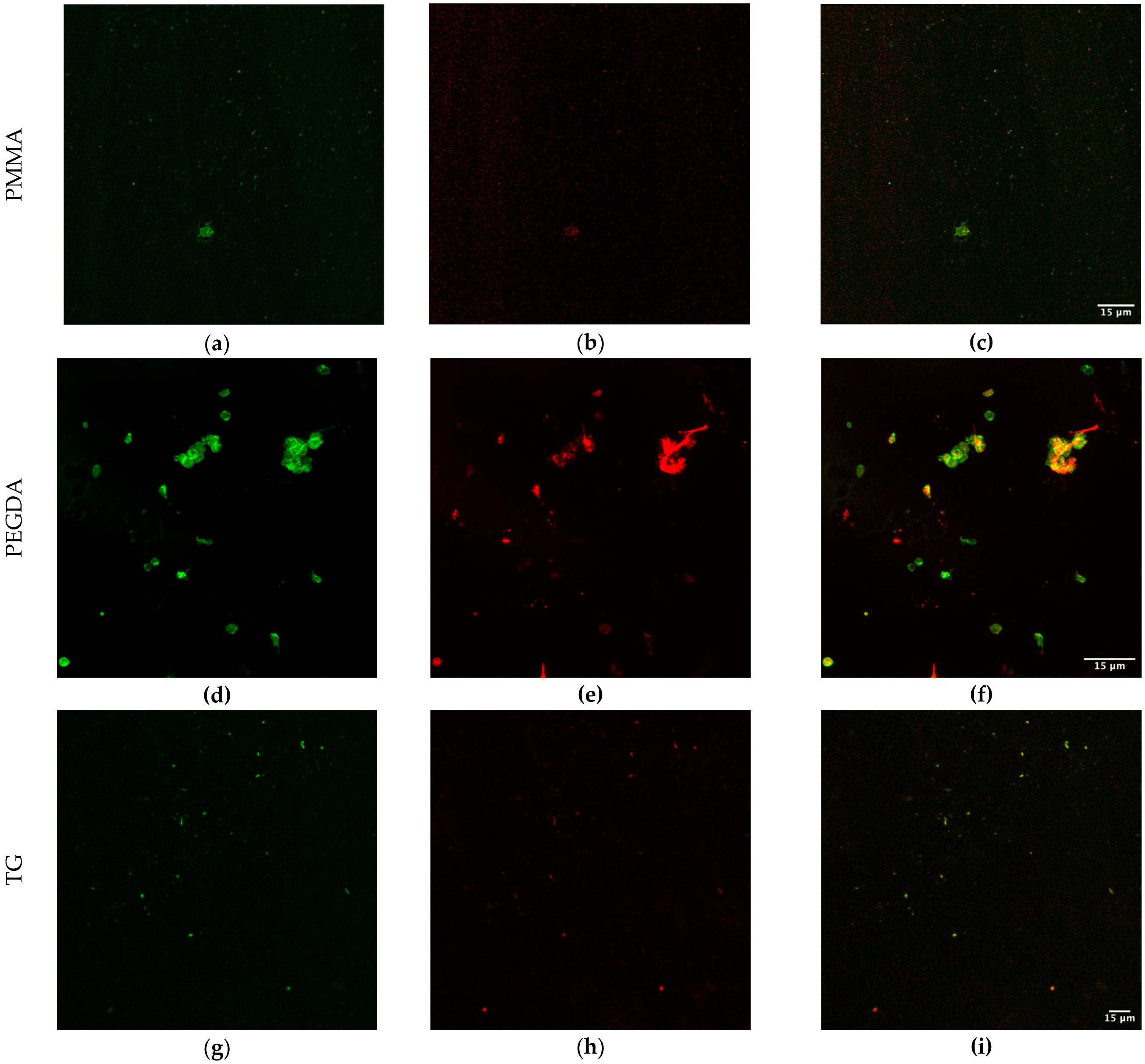

(**a**) (**b**) (**c**) (**d**) (**e**) (**f**) (**g**) (**h**) (**i**)

Figure S1. Confocal images of platelets adherent to polymeric materials. In the first column, the signal of CD41 is reported (panels a, d, g), while in the second the signal of CD62P is shown (panels b, e, h). Last column refers to merged signal (co-localization, panels c, f, i). Scale bars: 15 µm.

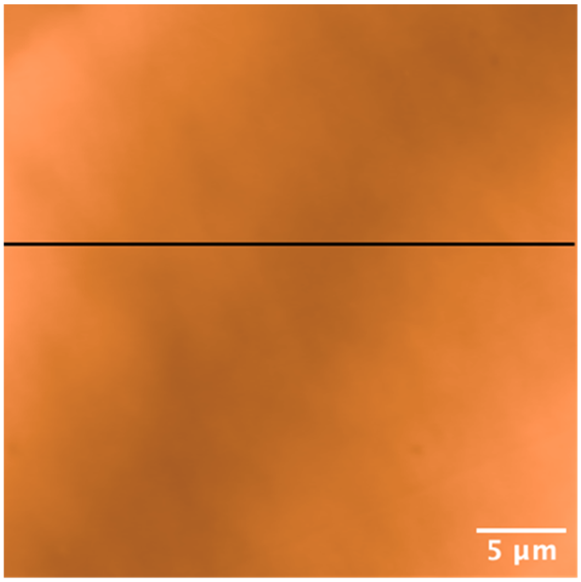

(a)

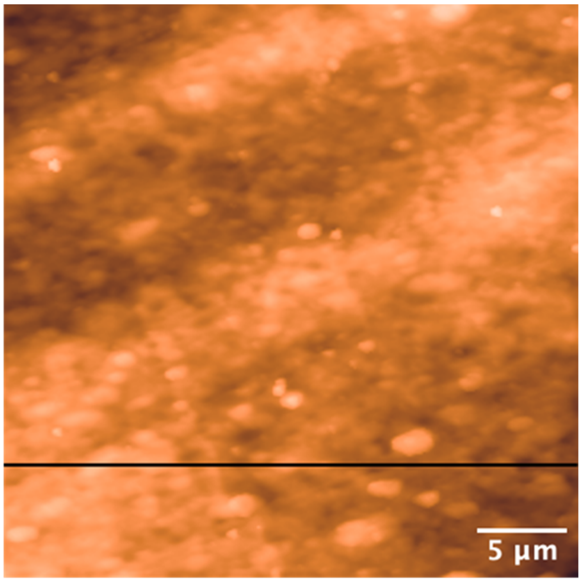

(b)

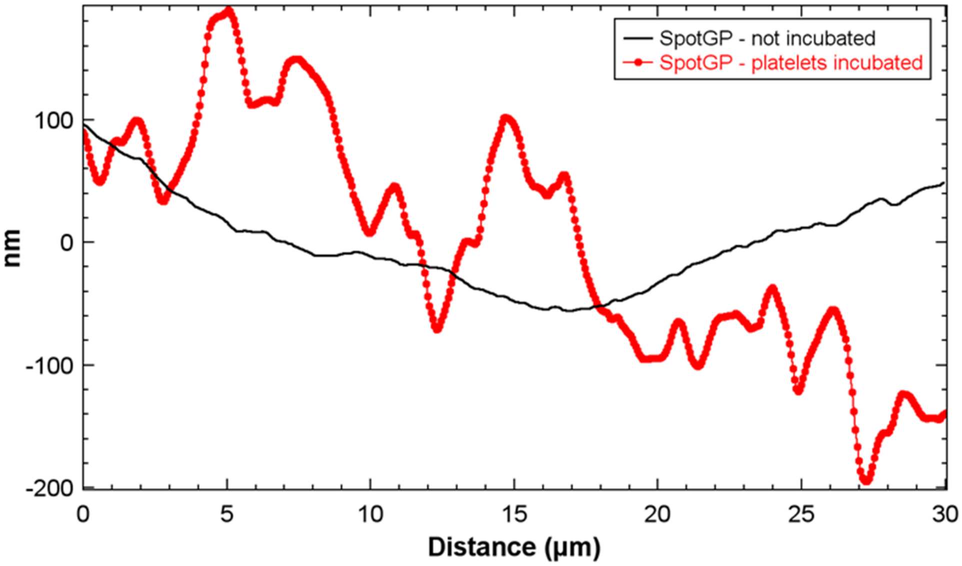

(c)

Figure S2. Typical AFM images of (a): untreated SpotGP, (b) SpotGP after platelets incubation. False color scale from -300 to 400 nm. Panel (c): height profiles traced along the black lines of (a) and (b).

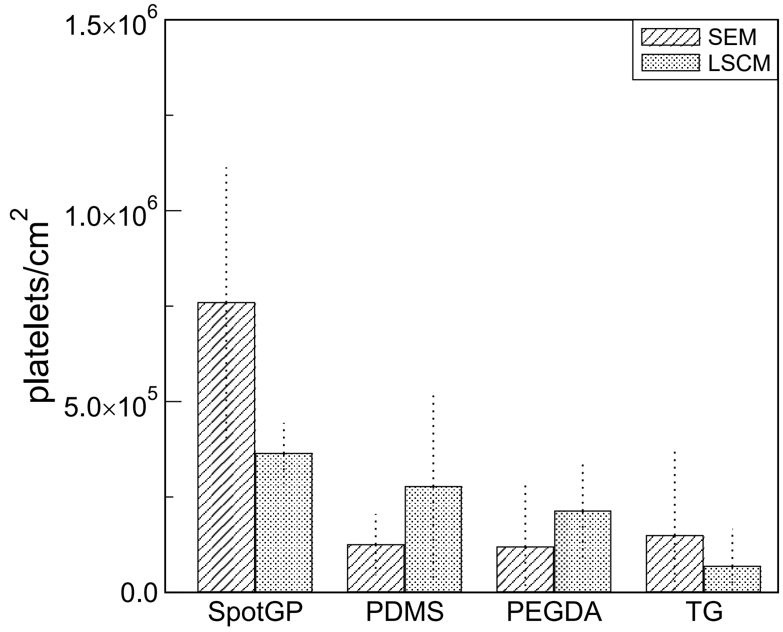

Figure S3. Comparison of platelet densities observed with Scanning Electron Microscopy and Laser Scanning Confocal Microscopy. Data are means of several images and standard deviations are shown.

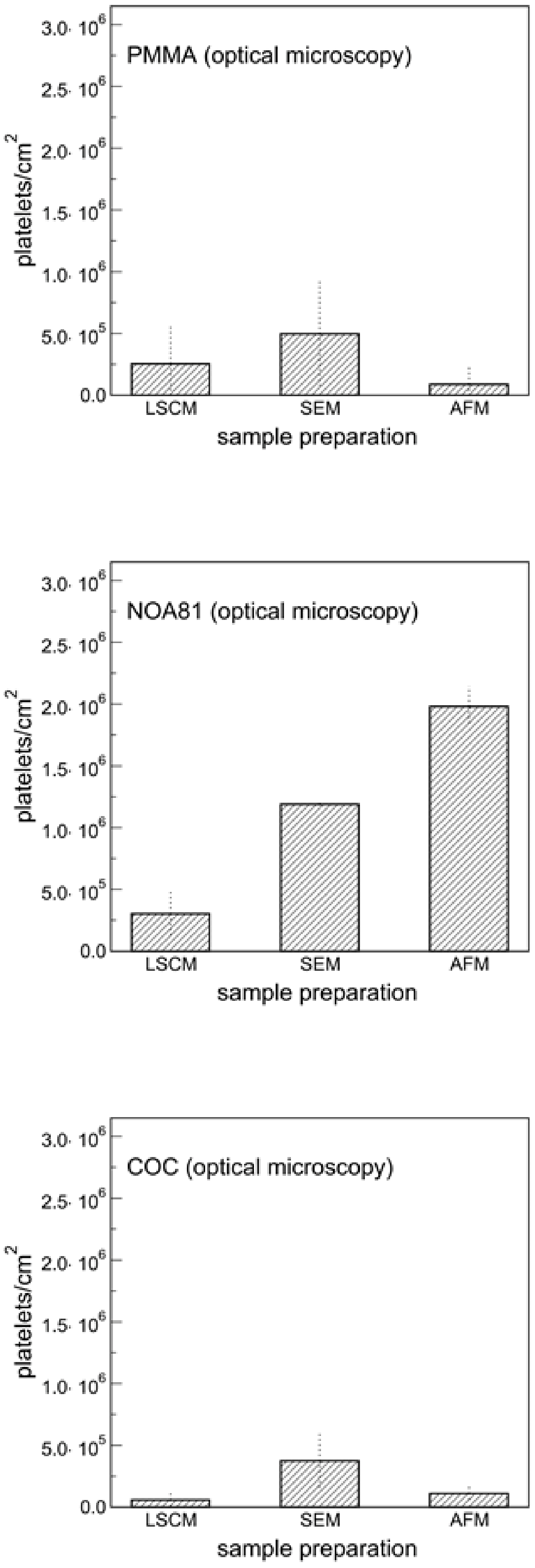

Figure S4. Comparison of platelet densities obtained with different sample preparations. Platelets prepared for LSCM, SEM or AFM imaging were measured by optical microscopy. Data are means of several images and standard deviations are shown.
